# COMMUNITY-RECRUITED OLDER ADULTS DIFFER FROM PATIENT POPULATIONS

**DOI:** 10.1093/geroni/igac059.3017

**Published:** 2022-12-20

**Authors:** Catherine Striley, Piyush Chaudhari, Roger Fillingim, Linda Cottler

**Affiliations:** University of Florida, Gainesville, Florida, United States; University of Florida, Gainesville, Florida, United States; University of Florida, Gainesville, Florida, United States; University of Florida, Gainesville, Florida, United States

## Abstract

Researchers often conduct randomized controlled trials among patient populations that may not reflect the community in which findings will be translated. The University of Florida’s community engagement program HealthStreet provides a diverse sample in which to consider differences between people 65 years of age and older who have seen a physician in the past 12 months and those who have not. This provides a conservative test of potential biases in patient-only samples. Based on a sample of 1,663 people, 65 years of age and older, who were recruited by Community Health Workers in the North Florida region from November 2011 through July 2022, 88% had seen a physician in the past 12 months. Those who had not (12%) were significantly more likely to be non-white than white, to be in good/excellent than fair/poor health, and significantly less likely to have a range of health conditions including high blood pressure, depression, heart conditions, diabetes, a digestive health condition, a dental health condition, or cancer. Yet residents who hadn’t seen a doctor were just as likely to be willing to participate in a future health study (both 94%) that only asked about health, accessed medical records (both 87%) or didn’t provide reimbursement (both 81%), and were not significantly different in attitudes toward participating in research in general. Recruiting of older adults should be conducted in the community, not just in patient populations, which are likely to be less diverse and sicker than those recruited through a community engagement program.

